# High-Throughput Sequencing Strategy for Microsatellite Genotyping Using Neotropical Fish as a Model

**DOI:** 10.3389/fgene.2018.00073

**Published:** 2018-03-09

**Authors:** Juliana S. M. Pimentel, Anderson O. Carmo, Izinara C. Rosse, Ana P. V. Martins, Sandra Ludwig, Susanne Facchin, Adriana H. Pereira, Pedro F. P. Brandão-Dias, Nazaré L. Abreu, Evanguedes Kalapothakis

**Affiliations:** ^1^Laboratory of Biotechnology and Molecular Markers, Department of General Biology, Institute of Biological Sciences, Federal University of Minas Gerais, Belo Horizonte, Brazil; ^2^Department of Zoology, Institute of Biological Sciences, Federal University of Minas Gerais, Belo Horizonte, Brazil

**Keywords:** microsatellite, fish, genotyping, next-generation sequencing, conservation genetics

## Abstract

Genetic diversity and population studies are essential for conservation and wildlife management programs. However, monitoring requires the analysis of multiple *loci* from many samples. These processes can be laborious and expensive. The choice of microsatellites and PCR calibration for genotyping are particularly daunting. Here we optimized a low-cost genotyping method using multiple microsatellite *loci* for simultaneous genotyping of up to 384 samples using next-generation sequencing (NGS). We designed primers with adapters to the combinatorial barcoding amplicon library and sequenced samples by MiSeq. Next, we adapted a bioinformatics pipeline for genotyping microsatellites based on read-length and sequence content. Using primer pairs for eight microsatellite *loci* from the fish *Prochilodus costatus*, we amplified, sequenced, and analyzed the DNA of 96, 288, or 384 individuals for allele detection. The most cost-effective methodology was a pseudo-multiplex reaction using a low-throughput kit of 1 M reads (Nano) for 384 DNA samples. We observed an average of 325 reads per individual per *locus* when genotyping eight *loci*. Assuming a minimum requirement of 10 reads per *loci*, two to four times more *loci* could be tested in each run, depending on the quality of the PCR reaction of each *locus*. In conclusion, we present a novel method for microsatellite genotyping using Illumina combinatorial barcoding that dispenses exhaustive PCR calibrations, since non-specific amplicons can be eliminated by bioinformatics analyses. This methodology rapidly provides genotyping data and is therefore a promising development for large-scale conservation-genetics studies.

## Introduction

Innovative technological applications in the field of conservation genetics can contribute to wildlife monitoring and management programs. Technological advances in genomics have greatly expanded the use of genetic markers in biological research, resulting in more extensive and efficient generation and analysis of population genetics data (Putman and Carbone, 2014).

Microsatellites are DNA units composed of repeating motifs in tandem, also known as simple sequence repeats (SSRs) or short tandem repeats. Due to their high degree of polymorphism, microsatellites are used as molecular markers in genetic structure, kinship identification, genetic mapping, and others population genetics studies (Buschiazzo and Gemmell, 2006; Chistiakov et al., 2006; Yazbeck and Kalapothakis, 2007; Bhargava and Fuentes, 2010). The high statistical power per *locus* obtained through microsatellites genotyping makes this a powerful tool in population studies (Guichoux et al., 2011; Putman and Carbone, 2014). Moreover, microsatellites are preferred in forensic and kinship analyses due to their high mutation rates and multiallelic nature (Clayton et al., 1998), and are the markers most frequently used in human paternity tests (Guichoux et al., 2011).

The early difficulties in isolating sequences from microsatellites were circumvented with the use of next-generation sequencing (NGS). Indeed, thousands of microsatellite *loci* can now be identified from a single NGS run (Tang et al., 2008; Boomer and Stow, 2010; Castoe et al., 2010; Rosazlina et al., 2015). However, the current techniques present difficulties regarding PCR calibration and the choice of informative microsatellites with high specificity. Thus, a balanced reaction that maintains great sensitivity and specificity to target DNA remains a challenge. In addition, studies involving microsatellites frequently use electrophoresis, which makes PCR calibration and fragment size identification laborious, and compromises the analysis of homoplasy cases (Delmotte et al., 2001; Pasqualotto et al., 2007).

NGS has enabled high-throughput genotyping, with a range of protocols for partial sequencing of genomes such as the use of restriction enzymes, e.g., RAD (Baird et al., 2008), ddRAD (Peterson et al., 2012), and 2bRAD (Wang et al., 2012). This is collectively known as ‘genotyping-by-sequencing’ (GBS) (Narum et al., 2013). Sequencing of a target region (PCR amplicon) can also be used as a GBS method (Vartia et al., 2015).GBS enables analyses of multiple target sequences in several different samples simultaneously, thereby saving time and resources. Moreover, target region sequencing can be applied to DNA samples with a certain degree of degradation such as forensic and cancer biopsy samples (Kerick et al., 2011; Van Neste et al., 2012; König et al., 2015). New approaches with microsatellite genotyping using individual combinatorial barcoding have been used in conservation genetics (Scheible et al., 2011; Vartia et al., 2015).

Here, we optimized a high-throughput genotyping methodology for genetic studies in conservation programs. We genotyped microsatellite *loci* employing NGS technology using *Prochilodus costatus*, a migratory fish species found in Brazil, as a model. Microsatellite markers with a maximum size of 200 base pairs (bp) and tri- or tetra-nucleotide motifs were used to facilitate data analysis (de Valk et al., 2005). We tested distinct NGS reagent kits and varying numbers of individuals per analysis. We believe that the use of high-throughput technology in conservation studies will advance the field, allowing large amounts of data to be generated quickly and efficiently.

## Materials and Methods

### Ethics Statement

We collected *P. costatus* samples in the region under the influence of the Três Marias dam (located in the state of Minas Gerais, Southeastern Brazil). To collect the samples needed for the study we obtained a Permanent Field Permit for Collecting from the Instituto Chico Mendes de Conservação da Biodiversidade (protocol number 57204-1) and also from the Instituto Estadual de Florestas (protocol number 014.007 2017).

### DNA Extraction

Using a sterile metallic mold, we removed 12.5-mm^2^ fragments of fish fin from samples previously collected and stored in 70% ethanol (v/v). Fragments were washed with ultrapure water and individually placed in 96-well plates. We added 50 μL of NaOH (50 mM) to the wells, sealed the plates, vortexed for 10 s, incubated at 95°C for 10 min, and vortexed again for 10 s. Then, 7.5 μL of Tris-HCl (0.5 M, pH 8.0) were added to each well and the plates vortexed for 15 s. Supernatants were transferred to new 96-well plates. We purified DNA from the supernatants using Agencourt AMPure XP magnetic beads (Beckman Coulter, Brea, CA, United States), according to the manufacturer’s protocol. DNA was quantified using Qubit 2.0 (Invitrogen, Carlsbad, CA, United States) and its purity was evaluated using NanoDrop 2000 (ThermoScientific, Waltham, MA, United States).

### Analysis and Identification of Microsatellites

Contigs previously generated by shotgun genome sequencing of *P. costatus* and deposited in the database of the Laboratory of Biotechnology and Molecular Markers were used for *in silico* analysis. The software msatcommander (Faircloth, 2008) was used to identify microsatellite regions. Over 100,000 contigs containing microsatellite regions were identified, and about 1,000 primer pairs suggested. We screened the primer pairs for the generated fragment size (amplicons of 130–200 bp), and selected microsatellites containing at least seven repeats of tri- or tetra-nucleotide motifs. de Valk et al. (2005) reported that microsatellites with tri- and tetra-nucleotide motifs can be easily discerned, avoiding issues such as stutter patter.

**FIGURE 1:**
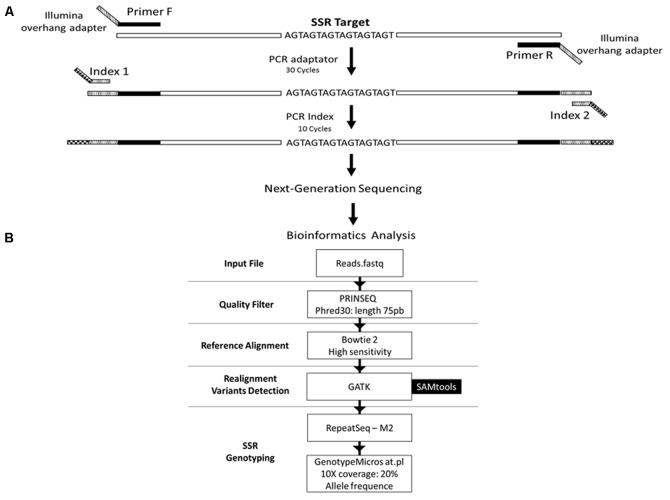
Schematic representation of the genotyping strategy. **(A)** Amplification protocol for generation of the amplicons for sequencing. **(B)** Data analysis pipeline. The reads from the sequencing are filtered based on the quality of the bases and the length of the read, using the PRINSEQ tool. Filtered reads are aligned against the reference sequence using the software Bowtie2 with high sensitivity parameter. Then, the reads are realigned against the reference, using tools from the GATK package, to increase the confidence in genotyping. Subsequently, alleles are detected and quantified using the RepeatSeq tool. Results are filtered to decrease the false negative rate using the GenotypeMicrosat.pl script. Finally, the result of the genotyping is converted into the format required by population genetics analysis software using the same script.

We selected 50 primer pairs that matched the requirements and tested them in *P.* *costatus* samples. PCR optimization was carried out by testing buffers with distinct concentrations of Mg^2+^ and KCl (Phoneutria Biotecnologia e Serviços LTDA, Brazil). PCR was performed using different annealing temperatures (50 – 65°C), dimethyl sulfoxide concentrations (0 – 7%), and numbers of amplification cycles (20 – 35). Amplicons were analyzed in 8% (w/v) polyacrylamide gel stained with silver nitrate. Twenty-four sets of primers were selected based on the presence of a single band (homozygous) or two adjacent bands (heterozygous). For genotyping, we used Illumina’s metagenomics protocol for amplicon resequencing. An ‘overhang adapter’ complementary to the ‘index’ in Illumina’s Nextera kits was added to the primers. The ‘overhang adapter’ sequences were as follows: Forward: 5′-TCGTCGGCAGC GTCAGATGTGTATAAGAGACAG + *locus*-specific forward primer sequence-3′, and Reverse: 5′-GTCTCGTGGGCTCG GAGATG TGTATAAGAGACAG + *locus*-specific reverse primer sequence-3′. The primers with the overhang adapters were incorporated into the target DNA through 30 PCR cycles. A specific primer (index) containing the MiSeq adapter (which individualizes samples in the NGS procedures) was attached to amplicons through 10 PCR amplification cycles (**Figure 1A**). A preliminary genotyping test (NGS sequencing) was performed using *P. costatus* DNA isolated from five individuals. From the 24 sets of primers, eight were selected for further analyses (**Table 1**). These were selected based on performance and PCR robustness (i.e., satisfactory amplification in non-ideal conditions, such as little or degraded DNA, the presence of inhibitors, etc.) and the presence of homozygous or heterozygous alleles with low amounts of unspecific DNA. GenBank accession number: Proc10 MG456705; Proc18 MG456707; Proc22 MG456708; Proc36 MG456709; Proc37 MG456710; Proc44 MG456712; Proc48 MG456715; Proc49 MG456716.

**Table 1:** Primers used for amplification of the microsatellite markers selected for genotyping of *Prochilodus costatus*.

Name	Primer F	Primer R	SSR Motif	Amplicon size (bp)	Buffer^∗^	AT (°C)	DMSO (%)	Number of Cycles
ProC10	ATTCCTGTCAATTTCCGGCC	AGGCCCAAACAGAAGGTAGG	ATT	130	4B	58	3	30
ProC18	GATCAGACCTCAGACGGGAC	GTTGTACGGAGATGCACTGC	CCGT	197	1C	60	5	25
ProC22	AGAGCTGGGATAGGCTCCAGC	CCTGGACAGGCTCCCAGTCC	AATG	130	4B	64	7	25
ProC36	GACGGAACGTCTTTAGAACC	TCTGCACATGCACGAGCGCGG	AAG	167	4B	62	5	30
ProC37	TGAAGGTGCACAGGGATAGT	TGTGAAGGTCCTGGAACCCAC	TTTA	130	4B	62	–	25
ProC44	CTTAGTGAACTGGAGCACG	GGTCCAGATTGGGCATATACAC	AAC	183	1C/4B	58	–	25
ProC48	AGCTTAGATGTGTACTAAC	GTTGAGCAGTGGTGGGGTAC	AAAG	153	1C	55	–	25
ProC49	GGTGTTTGGTTAATCACCCC	AGAGATGTGCTTATGCACGC	AAAG	146	4B	62	7	25

*SSR, simple sequence repeat; bp, base pairs; AT, annealing temperature; DMSO, dimethyl sulfoxide. ^∗^Buffer classification according to Phoneutria Biotecnologia e Seviços LTDA, Brazil*.

We tested several NGS genotyping strategies: (a) multiplex, in which all primer pairs were used to amplify a single DNA sample in a single reaction (30 cycles) followed by the incorporation of the index (10 cycles) and NGS sequencing; (b) pseudo-multiplex reaction, in which each primer pair was individually used to amplify a DNA target (30 cycles), followed by pooling the amplicons of one individual and a second PCR (10 cycles) for index incorporation and MiSeq sequencing; (c) monoplex, in which DNA target amplification (30 cycles) and index incorporation (10 cycles) were performed individually for each DNA target and followed by MiSeq sequencing. **Table 2** indicates the strategies, the types of cartridges, and the number of target DNA samples used (minimum of five and maximum of 384).

**Table 2:** Strategies used for optimization of next generation sequencing (NGS) microsatellite genotyping of *Prochilodus costatus*.

NGS Run	Strategy^#^	N° of primer pairs	N° of DNA samples	Cartridge^¥^	N° of filtered reads	^∗∗^N° of alleles
1	Multiplex	8, 4 and 2	5	1 M	13,691	^∗^
	Monoplex	8			829,631	^∗^
2	Multiplex	8	192	15 M	608,390	30
3	Pseudo-multiplex	8	192	1 M	486,130	49
4	Pseudo-multiplex	8	96	1 M	646,223	48
5	Pseudo-multiplex	8	288	1 M	1,051,375	52
6	Pseudo-multiplex	8	384	1 M	991,337	53

^∗^*Not applicate due to the low number of individuals. ^∗∗^Total number of alleles obtained for eight *loci* and variable numbers of individuals. ^#^Reactions used for amplification. Multiplex, in which all primer pairs were used to amplify a single DNA sample in a single reaction followed by the incorporation of the index and NGS sequencing; Pseudo-multiplex, in which each primer pair was individually used to amplify a DNA target, followed by pooling of the amplicons of one individual (eight *loci* per individual) and a second PCR for index incorporation and MiSeq sequencing; Monoplex, in which DNA target amplification and index incorporation were performed individually for each DNA target and followed by MiSeq sequencing. ^¥^Type of cartridge used. 1 M is a MiSeq Reagent Nano Kit v2 (1 M reads) and 15 M is a MiSeq Reagent Kit v2 (15 M reads)*.

To optimize the NGS genotyping procedures, we performed multiplex PCR tests in an initial MiSeq run (run 1) using a low-throughput kit (1 M reads, MiSeq Reagent Kit Nano, 300 cycles; Illumina). We mixed eight, four, and two primer pairs for the first, second, and third tests, respectively. Additionally, a monoplex test was carried out for each of the eight primer pairs selected. We used DNA from five individuals in all tests (**Table 2**) and different index sequences for each test.

The second NGS run (run 2) was carried out using a multiplex reaction with the eight selected primer pairs, 192 individuals, and a high-throughput kit (15 M cartridge, MiSeq Reagent Kit Standard, 300 cycles; Illumina). Pseudo-multiplex reactions with eight primer pairs and 1M cartridges were performed in the runs 3, 4, 5, and 6 with 192, 96, 288, and 384 target DNAs, respectively (**Table 2**).

We used Nextera XT Index Kit v2 Sets A, B, C, and D (Illumina), which allows the sequencing of up to 384 individuals in a single MiSeq run and, therefore, accelerates the process and increases the benefit-cost ratios of the analysis. We further increased the number of individuals genotyped per run using a random nucleotide sequence (such as AAA or TTT) between the adapter and the *locus*-specific sequences. We used the following primers: Forward: 5′-TCGTCGGCAG CGTCAGATGT GTATAAGAGA CAG + **AAA** or **TTT** + *locus*-specific forward primer sequence-3′, and Reverse: 5′-GTCTCGTGGGCTCGGAGATG TGTATAAGAG ACAG + **AAA** or **TTT** + *locus*-specific reverse primer sequence-3′ (see **Table 1** for primer sequences). In these runs, only one set of adapters was used (Nextera XT Index kit v2 set A) and 288 individuals were genotyped. Noteworthy, other sequences can be used including CCC or sequences with four or more nucleotides. However, shorter sequences (one or two nucleotides) may hinder bioinformatics analysis, whereas longer sequences may reduce the efficiency of amplification. Additional tests should be performed to evaluate each case.

Amplicons of the eight *loci* were polled for each individual, quantified using Qubit, and diluted to 10 ng/μl. Then, a new pool (the combinatorial barcoding amplicon library) was prepared with all the quantified material. This library was then quantified by qPCR using the KAPA Library Quant Illumina/Universal kit (KAPA Biosciences) following the manufacturers’ instructions. The library was used as input in a MiSeq run with a final concentration of 15 pM. A ready-to-use control library from Illumina (PhiX Control v3) was used in each sequencing run.

### Genotyping of Microsatellites

We developed a bioinformatics pipeline for microsatellite genotyping (described below and in **Figure 1B**). To improve reliability, we trimmed and filtered the obtained reads using the PRINSEQ tool (Schmieder and Edwards, 2011). Bases with Phred scores lower than 30 and/or read lengths shorter than 75 bp were removed. Filtered reads were aligned against a FASTA file containing reference sequences for the eight microsatellite *loci* using the software Bowtie 2 (Langmead and Salzberg, 2012) with the high sensitivity option.

Alignment against a reference region that contains insertions or deletions of nucleotides, such as the microsatellite variants requires careful curation because variations in the edge of the repeat can lead to error in alignment and consequent misidentification of the alleles. One way to increase the confidence and reduce error is to realign the reads taking into account the nucleotide sequence of the edges of the repeat and possible variants within the region. As no information on the variants of the microsatellite region studied here was available in public databases, we identified the possible variants at the edges of the repetition of each of the eight *loci*. We used the SAMtools package (Li et al., 2009) to detect possible variants in the mapping file (BAM format) of each of the 384 sequenced individuals. As a result, we obtained a Variant Call Format (VCF) file containing all the variants in the regions around the microsatellites repeat motifs. Next, we realigned the reads that mapped to the reference repeating regions using the tools RealignTargetCreator and IndelRealigner from the GATK package (McKenna et al., 2010). These tools perform a local realignment to the regions of the repeat motifs taking into account only high-quality reads that completely cover the repeat region and the variants described for the region (VCF file). Reads that did not match these criteria were removed. A realignment file (BAM format) containing only reads that realigned to each *locus* (tested twice) was obtained for each individual, thus increasing the confidence in the identification of the alleles.

We used the RepeatSeq tool (Highnam et al., 2012), with parameter -M 2 (minimum sequencing quality required value) to identify and quantify the alleles from the realignment files. This tool requires a file containing the chromosome coordinates and the repeat region motif sequence. Since *P. costatus* genomic information was unavailable, we used the information obtained through the microsatellites amplicon sequencing as an independent chromosome. We created an input file containing the name, the starting and ending positions of the repeat sequence in the amplicon, and the sequence of repeat motif for each *locus*. The information of the chromosomal regions was replaced by the information of each of the amplicons. There is no limit regarding the size or number of amplicons. However, it is important to enter the correct location and base sequence of the repeat region. The RepeatSeq tool uses the coordinate file to search for repeat regions in the realignment files and calculates the repeat length, which determines the alleles. A repeat ATTATTATTATT, for example, would be defined as allele 12. After identification of the repeat motif, the reads that aligned to that region are selected and quantified, according to their number of repeats. The resulting file contains the full read annotation of the reference microsatellite, including the total number of alleles detected, total number of reads, total number of reads per allele, and mapping quality score.

To avoid false negatives and to convert the results into the input format required by the software commonly used in population genetics, we developed a Perl script, named GenotypeMicrosat.pl. This script performs a detailed analysis of the RepeatSeq output file. We determined the individual’s genotype for each *locus* using the following filter criteria: (1) maximum of two alleles per individual per *locus*; (2) at least 10 reads per *locus* in the entire repeat sequence, including eight bases in the 5′ and eight bases in the 3′ flanking regions; and (3) at least 20% of reads corresponding to a second allele for an individual to be considered heterozygous in a given *locus*. Individuals with a second allele coverage of less than 20% were considered homozygous. Following application of these filter criteria, we generated a spreadsheet containing the genotypes of each individual for each of the eight *loci*. *Loci* that did not attain the filter requirements were identified as ‘NA.’ The generated spreadsheet can be easily adapted for other population genetics analysis programs.

## Results

**FIGURE 2:**
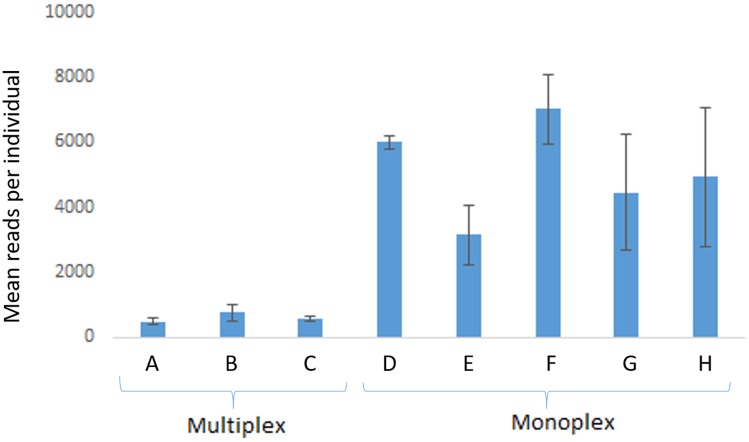
Average (mean) read yield in run 1 (*n* = 5 individuals). Multiplex reactions were performed in A with eight *loci* (proC10, proC18, proC22, proC36, proC37, proC44, proC48, proC49), in B with four *loci* (proC18, proC36, proC37, proC44), and in C with two *loci* (proC48 and proC49). Monoplex reactions were performed in D (proC10), E (proC18), F (proC36), G (proC48), and H (proC49). Error bars are standard deviation. MiSeq Reagent Kit v2 1 M reads was used.

In comparison with monoplex, multiplex reactions generated 10- to 100-times fewer reads (run 1) that could be used in genotyping (**Figure 2**). For some *loci*, no reads were detected in the multiplex tests, which suggests intense primer competition. On the other hand, consistent results were observed with monoplex-amplified samples (**Figure 2**).

We compared the genotyping results of 192 individuals by multiplex (run 2) or pseudo-multiplex (run 3) reactions. The 15 M cartridge tested in run 2 yielded about 4.7 times more reads than the 1 M cartridge (Nano) kit tested in run 3. Nevertheless, the number of reads generated with the Nano kit (run 3) allowed genotyping of all individuals. In run 2 (multiplex, **Table 3**), reads were obtained for five *loci* only. Amplification efficiency, determined as the fraction of individuals successfully genotyped for a given *locus*, was superior in the pseudo-multiplex reaction (run 3) for all *loci*, except proC10 and proC37 (**Table 3**).

**Table 3:** Amplification and genotyping efficiency test for multiplex (run 2) and pseudo-multiplex (run 3) systems.

*Loci*	Multiplex (%)	Pseudo-multiplex (%)
ProC10	97.86	86.09
ProC18	X	70.05
ProC22	44.92	80.21
ProC36	50.27	93.05
ProC37	50.80	16.04
ProC44	19.79	65.77
ProC48	X	63.64
ProC49	X	97.86

*Values correspond to the genotyping efficiency (%) for 192 individuals (*Prochilodus costatus*). x, no date obtained*.

**FIGURE 3:**
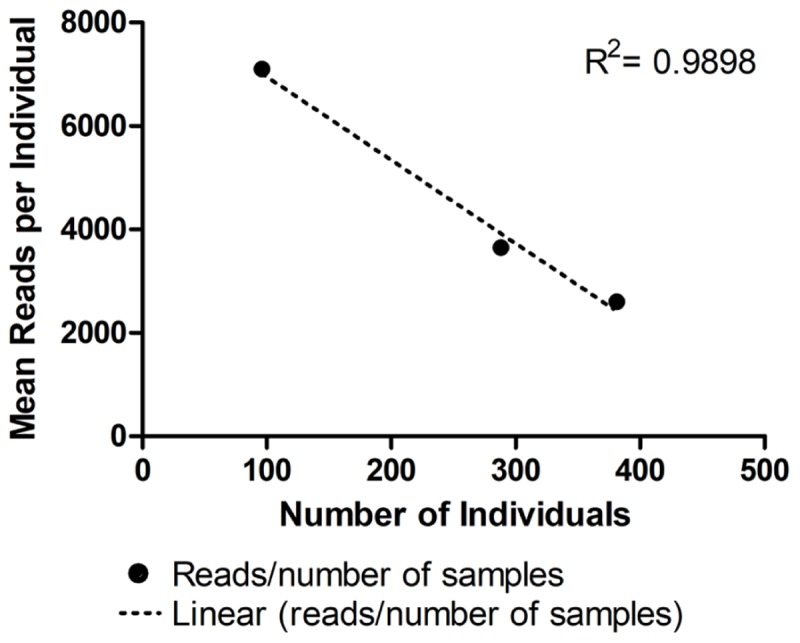
Average number of reads generated per individual in three distinct runs using MiSeq Reagent Kit v2 1 M reads: run 4 (96 individuals), run 5 (288 individuals), and run 6 (384 individuals). • Represents the mean number of reads for each number of samples tested. Linear regression is represented by the dashed line (^……^). For more details, see **Table 2**.

As expected, the standard curve generated from runs 4, 5, and 6 revealed a strong negative correlation between the number of individuals tested and the number of reads generated (**Figure 3**). The obtained distribution of reads per *locus* in these three runs (**Table 4**) was uneven. When analyzing 384 individuals (run 6), we encountered an average of 325 reads per individual per *locus* for all *loci*. The yield differed depending on the *locus* being evaluated and for the same *locus* in different runs. For example, marker proC36 yielded an average of 1,018 reads per individual in run 5 and 198 reads per individual in run 6. On the other hand, proC37 yielded only 65 and 42 reads in runs 5 and 6, respectively. This variation did not compromise the genotyping analysis because of the bioinformatics parameters used.

**Table 4:** Percentage^∗^ of reads generated for each *locus* tested in three distinct sequencing runs (runs 4, 5, and 6).

*Loci*	% run 4	% run 5	% run 6
ProC10	5.79	10.36	5.44
ProC18	5.50	10.66	3.01
ProC22	12.69	8.08	14.52
ProC36	6.39	27.89	19.02
ProC37	0.26	1.78	0.21
ProC44	4.49	6.26	2.49
ProC48	7.32	3.99	3.81
ProC49	57.56	30.99	51.50

^∗^*Percentage was calculated from 646,223 reads generated in run 4 with 96 individuals, 1,051,375 reads generated in run 5 with 288 individuals, and 991,337 reads generated in run 6 with 384 individuals. One MiSeq Reagent Nano Kit v2 (1 M reads) was used for each run*.

From the 3,771,786 reads yielded in run 6, 1,998,885 (53%) passed quality filtering. Repeatseq identified 991,337 high-quality reads (**Table 2**), which were subsequently used for allele detection. Of the 3,072 microsatellites genotyped in this run (eight *loci* from 384 individuals), 1,179 (39%) remained with undetermined genotype due to the low number of reads per *locus* (<10). The total number of alleles obtained in run 6 (384 individuals and 1 M cartridge) was 53, with the *locus* proC49 showing the highest number of alleles (10) while proC10 showed the lowest (4). Genotyping using the primers marked with base triads AAA and TTT generated results similar to those obtained in run 6.

## Discussion

In the present paper we showed the potential of microsatellite genotyping by NGS as a fast and cost-effective methodology to be implemented in large-scale population genetic studies. We used a combination of commercially available indexes and genotyped 384 individuals per run. The efficiency observed with the Nano (1 M reads) kit represents a substantial cost reduction over the NGS runs with the 15 M reads kit.

The flowchart presented herein was developed to ensure high accuracy in microsatellite genotyping. We excluded low-quality reads from the analysis and aligned the reads against the reference using Bowtie 2, the best-suited software for INDEL-rich *loci* (Highnam et al., 2012). This realignment step increases the confidence in allele detection, since our analysis considers only reads that cover the whole repeat region, including eight bases in the 5′ and eight bases in the 3′ flanking regions. Additionally, the pipeline proposed verifies all neighboring variations (5′ and 3′ regions flanking the microsatellite motif), allowing the identification of homoplastic motif repeat numbers and fragment length. The microsatellite genotyping tool RepeatSeq uses a Bayesian approach, which considers characteristics of the read and the sequence under analysis (Highnam et al., 2012). The developed GenotypeMicrosat.pl script further increases the confidence of the genotyping by establishing a minimum of 10 reads to confirm an allele.

Read realignment with the GATK package requires a file containing all the variants described for the analyzed species. However, information about variations in our model species is scarce in public databases. As an alternative, we used single nucleotide polymorphisms (SNPs) and INDELs detected through sequenced amplicons as input for GATK. To circumvent the lack of data on the *P. costatus* microsatellite genomic localization required by RepeatSeq, we generated a file containing the genomic coordinates and repeat sequences to use as input. Our successful attempts to overcome the lack of genomic information for our species of interest highlight the potential application of the pipeline proposed for microsatellite genotyping of species for which genomic data are not available, and further support its use in genetic monitoring programs.

The maximum number of individuals genotyped per run is limited by the number of commercially available indexes (currently 384). However, the amplification success of primers containing adenine or thymine trios shows the potential of this tool to increase the number of individuals genotyped in a single run. We genotyped eight *loci* per individual, with an average coverage of 325 reads per *locus* per individual in the runs with the Nano kit using 384 samples. As the genotyping pipeline considers a minimum of 10 reads per allele per individual, none of the runs reached the maximum capacity of the cartridge. In theory, the number of individuals or the number of *loci* could be increased up to four times per run (32 *loci* or 1536 individuals), based on the regression analysis. However, this possibility must be weighted carefully since the number of alleles considerably varies among the *loci*. For instance, ProC49 showed an average of 700 reads, while ProC37 showed 20 reads per individual in run 6. The number of *loci* may, therefore, be increased or decreased depending on the quality of the *loci* tested. Previous knowledge of the quality of a given *locus* also allows for the use of greater amounts of amplicon for *loci* with low yield. These findings open the prospect of using *loci* for which PCR reactions are not 100% efficient and represent an advantage in the genetic analysis of understudied species with limited availability of microsatellites.

Traditional methods employing microsatellite molecular markers have disadvantages such as the long optimization process and the elevated costs, especially in the development of multiplex systems. Additionally, automation limitations and data management requirements can prevent technology transfer among different laboratories (Guichoux et al., 2011). Previous studies have shown that allele sizes generated by capillary electrophoresis may vary depending on the equipment and running conditions (Delmotte et al., 2001; Foulet et al., 2005; Pasqualotto et al., 2007), and the number of *loci* that can be multiplexed with this technique is limited by the number of commercially available fluorophores. On the other hand, many *loci* can be simultaneously genotyped in a single NGS run (Scheible et al., 2011). The direct sequencing of *loci* is a more reliable approach as it allows for the analysis of all the variations in the fragment, thus ensuring greater reliability. Furthermore, technology transfer and detection of technical errors are facilitated by NGS. Here we tested eight *loci* per individual. Nevertheless, our pipeline has the potential to provide analysis of a significantly larger number of *loci* and recent publications with neotropical migratory fish revealed a minimum of seven and a maximum of 13 *loci* for up to 30 individuals (Rueda et al., 2011; Berdugo and Narvaez Barandica, 2014; Coimbra et al., 2017).

Despite the poor results of direct multiplex reactions, we successfully optimized a ‘pseudo-multiplex strategy,’ in which previous monoplex reactions were performed for each sample and the amplicons mixed in the indexing reaction. This strategy reduced the cost and duration of the analysis and may be used in the genotyping of other markers, such as SNPs, and in metagenomics studies.

## Conclusion

We present a novel method for microsatellite genotyping based on the Illumina combinatorial barcoding using a Nano kit. This approach is faster and more efficient than those currently available and offers large amounts of high-quality data for conservation genetics and population studies.

## Author Contributions

JP and EK designed and coordinated the work. SL, SF, AP, PB-D, and NA acquired the data. AC, IR, and AM developed the pipeline. JP, AC, IR, and SL analyzed the data. All authors contributed to data interpretation and provided substantial contributions to manuscript writing. All authors approved the final version prior to submission.

## Conflict of Interest Statement

The authors declare that the research was conducted in the absence of any commercial or financial relationships that could be construed as a potential conflict of interest.
